# A randomised clinical trial (RCT) of a symbiotic mixture in patients with irritable bowel syndrome (IBS): effects on symptoms, colonic transit and quality of life

**DOI:** 10.1007/s00384-012-1552-1

**Published:** 2012-08-12

**Authors:** Carmelina Cappello, Fabrizio Tremolaterra, Annalisa Pascariello, Carolina Ciacci, Paola Iovino

**Affiliations:** 1 Chirurgia Generale e Geriatrica, Facoltà di Medicina e Chirurgia, Università degli Studi di Napoli Federico II, Via Pansini 5, 80131 Napoli, Italy; 2 Gastroenterologia, IRCCS-Centro di Riferimento Oncologico della Basilicata, Via Padre Pio 1, Rionero in Vulture, 85028 Potenza, Italy; 3 Gastroenterologia, Dipartimento di Medicina e Chirurgia, Università degli Studi di Salerno, Via S Allende, 84081 Baronissi, Salerno Italy

**Keywords:** Irritable bowel syndrome, Symbiotic, Probiotic, Prebiotic, Bloating, Flatulence, Randomised clinical trial

## Abstract

**Purpose:**

The aim of this study is to test in a double-blinded, randomised placebo-controlled study the effects of a commercially available multi-strain symbiotic mixture on symptoms, colonic transit and quality of life in irritable bowel syndrome (IBS) patients who meet Rome III criteria.

**Background:**

There is only one other double-blinded RCT on a single-strain symbiotic mixture in IBS.

**Methods:**

This is a double-blinded, randomised placebo-controlled study of a symbiotic mixture (Probinul, 5 g bid) over 4 weeks after 2 weeks of run-in. The primary endpoints were global satisfactory relief of abdominal flatulence and bloating. Responders were patients who reported at least 50 % of the weeks of treatment with global satisfactory relief. The secondary endpoints were change in abdominal bloating, flatulence, pain and urgency by a 100-mm visual analog scale, stool frequency and bowel functions on validated adjectival scales (Bristol Scale and sense of incomplete evacuation). Pre- and post-treatment colonic transit time (Metcalf) and quality of life (SF-36) were assessed.

**Results:**

Sixty-four IBS patients (symbiotic *n* = 32, 64 % females, mean age 38.7 ± 12.6 years) were studied. This symbiotic mixture reduced flatulence over a 4-week period of treatment (repeated-measures analysis of covariance, *p* < 0.05). Proportions of responders were not significantly different between groups. At the end of the treatment, a longer rectosigmoid transit time and a significant improvement in most SF-36 scores were observed in the symbiotic group.

**Conclusions:**

This symbiotic mixture has shown a beneficial effect in decreasing the severity of flatulence in IBS patients, a lack of adverse events and a good side-effect profile; however, it failed to achieve an improvement in global satisfactory relief of abdominal flatulence and bloating. Further studies are warranted.

## Introduction

Irritable bowel syndrome (IBS) is a common gastrointestinal disorder characterised by abdominal pain or discomfort and altered bowel habits [1]. The aetiology of IBS is still poorly understood, and different pathophysiological mechanisms have been proposed: alterations in gut motility, small-bowel bacterial overgrowth, microscopic inflammation, visceral hypersensitivity and changes related to the brain–gut axis [1]. On the basis of these different aetiopathological hypotheses, a variety of therapies have been tested in IBS patients to improve their symptoms without reaching a complete resolution [2].

In theory, certain probiotics, at a right dose and in the appropriate formulation, can help restore the proper balance of the intestinal microbiota, leading to a better digestive and intestinal function and possibly improve gastrointestinal symptoms [3]. A mixture of probiotics and prebiotics, namely symbiotic, should exert a synergistic benefit by the enhancement of the probiotic organisms by the selective, coadministered prebiotic substrate [4].

Most of the previous studies testing the effects of probiotics in IBS showed mixed results largely related to methodological limitations, such as the use of different probiotic bacteria in each study and the use of heterogeneous and not clearly defined study population. However, despite considerable limitations, recently several randomised trials comparing the effects of probiotics vs placebo in IBS showed some interesting positive effects on many gastrointestinal symptoms, especially bloating and flatulence [5–8]. Taken together, the data coming from studies on probiotics in IBS patients demonstrated that not all probiotics are the same and that the effect of a specific probiotic cannot be extrapolated to another probiotic strain [9–11]. The mechanisms of such benefits are unfortunately still poorly understood. One of the hypotheses is supported by the clear recognition that IBS may in fact be induced by bacterial gastroenteritis (post-infectious IBS) [12] and that qualitative changes in the flora, as well as an immune dysfunction, may be more prevalent in IBS in general [13]. Furthermore, it has been shown that the interaction between commensal microbiota and the host might have an important role in the modulation of the gut sensory–motor function [14]. The rationale for choosing this symbiotic mixture for our study was based on several reasons: the product is already available on the market, has a record of safe human consumption and to our knowledge there is only one other double-blinded RCT on a single-strain symbiotic mixture which was performed regarding IBS [15]. Thus, the hypothesis of our study was that the beneficial effects of manipulation of the intestinal microbiota in patients with IBS, through a symbiotic mixture, can result in an improvement of abdominal symptoms, i.e. bloating and flatulence, a change in colon transit time and an improvement in the quality of life.

## Aim

Our aim was to test in a double-blinded, randomised placebo-controlled study the effects of a commercially available symbiotic mixture in IBS patients.

## Methods

### Study population

Sixty-four IBS patients were recruited from an outpatient clinic, which is only devoted to gastrointestinal functional disorders. The study protocol had previously been approved by the ethical committee of Federico II University of Naples and therefore had been performed in accordance with the ethical standards laid down in the 1964 Declaration of Helsinki. All participants signed a consent form prior to being enrolled in the study.

The diagnosis of IBS was made on the basis of the Rome III criteria [16] together with the exclusion of any other organic disease. The severity of IBS was scored using the validated Functional Bowel Disorders Severity Index (FBDSI), developed by Drossman et al., which provides an easy-to-use scale to appraise illness severity in this complex group of patients. It is comprised of three variables: current pain (by 0–100 visual analog scale (VAS)), diagnosis of chronic abdominal pain and the number of physician visits within the past 6 months. Severity was rated as mild (1–36 points), moderate (37–110 points) and severe (≥111 points). Details of the index calculation are presented in the original article [17].

The eligibility criteria included females and males between the ages of 18 and 75 having the ability to understand and the willingness to comply to the study procedures; females with childbearing potential were required to have a negative pregnancy blood test within 48 h of the two colonic transit studies. They were also required to have an average daily abdominal bloating score or flatulence ≥24 on a 100-mm VAS during the 2-week run-in period. Patients were excluded if they had serious, unstable medical condition, insulin-dependent diabetes mellitus, major psychiatric diagnosis, previous history of drug or alcohol abuse 6 months prior to screening, a diagnosis of lactase deficiency or if symptoms resolved with lactose-free diet and if they had undergone previous abdominal surgery except appendectomy, caesarean section, tubal ligation, laparoscopic cholecystectomy, hysterectomy or abdominal wall hernia repair. They were also excluded if they currently used medications that may alter gastrointestinal motility, or long-term antibiotics or proton pump inhibitors. Patients were also carefully interrogated on use of over-the-counter medication within 3 days of transit studies and antibiotic use 1 month prior to screening.

### Concomitant medications

The following medications were permitted during the course of the study, as long as they had been used at a constant dosage and were commenced at least 1 month prior to the start of the 2-week run-in period: birth control pill or depot intramuscular contraceptive preparation, oestrogen–progesterone replacement therapy, l-thyroxine, low-dose antidepressants (up to 25 mg day^−1^ of amitriptyline, nortriptyline or selective serotonin reuptake inhibitor) or antihypertensives in the diuretic, angiotensin-converting enzyme inhibitor or angiotensin II inhibitor classes.

### Study design

**Fig. 1:**
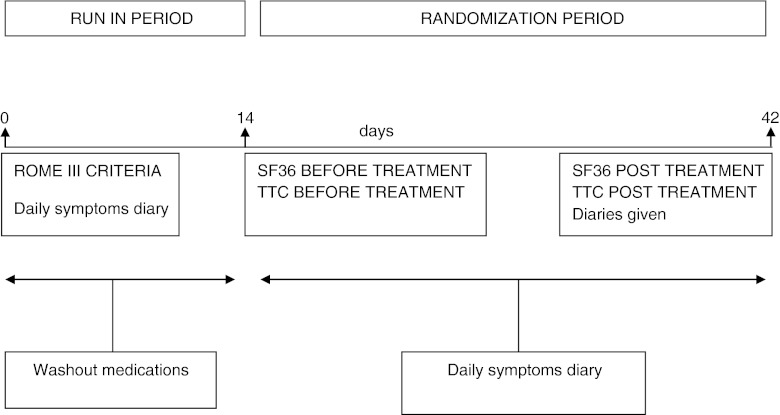
Schematic presentation of the study design

A parallel-group, double-blinded, placebo-controlled study with a total of 64 patients being randomised to either placebo (*n* = 32) or symbiotic (*n* = 32) was planned. Randomisation was carried out according to a computer-generated, blocked randomisation list independent of the research group (Statistical Department of Istituto Di Sanità, Roma) and with a concealed block size of 4. The patients, the investigators, the doctors and the study nurse were blinded using randomisation codes, which were kept confidential until the end of data analysis. Eligible patients were required to pursue a baseline 2-week run-in period recording daily symptoms by means of a diary and, followed by a 4-week treatment period (Fig. 1). All patients underwent colonic transit measurement and filled in the quality of life questionnaire at the end of run-in and of the treatment phase.

### Study treatments

The symbiotic mixture (Probinul, CaDi Group, Rome, Italy) contains lyophilised bacteria (5 × 10^9^ *Lactobacillus plantarum*, 2 × 10^9^ *Lactobacillus casei* subp. *rhamnosus* and 2 × 10^9^ *Lactobacillus gasseri*, 1 × 10^9^ *Bifidobacterium infantis* and 1 × 10^9^ *Bifidobacterium longum*, 1 × 10^9^ *Lactobacillus acidophilus*, 1 × 10^9^ *Lactobacillus salivarus* and 1 × 10^9^ *Lactobacillus sporogenes* and 5 × 10^9^ *Streptococcus termophilus*), prebiotic inulin (2.2 g; VB Beneo Synergy 1) and 1.3 g of tapioca-resistant starch. The study preparation was provided in a powder form (5-g sachets) containing the symbiotic mixture or the matching placebo (CaDi Group, Rome, Italy) comparable in colour, texture and taste to the symbiotic mixture. The patients were instructed to ingest the investigational powder preparation twice daily, preferably in the morning and in the evening far from meals, dissolving it in water.

### Assessment of symptoms and bowel functions

Patients filled in a daily diary to evaluate stool frequency, bowel function and symptoms. Bowel function was assessed on validated adjectival scales: stool consistency by Bristol stool form scale [18–21], and the sensation of incomplete evacuation was evaluated by a binomial answer (yes or no). Bowel symptoms were bloating, sensation of flatulence, pain and faecal urgency which were assessed on 100-mm VAS. Moreover, they tracked the intake of all medications during the trial. If they received antibiotics for acute infections (for example bacterial throat or urinary infections), the symptom data for the time of antibiotic administration and 1 week after completion were excluded from analysis and the treatment period was prolonged to ensure availability of the requisite number of weeks of study treatment. At the end of each week, patients were required to record a weekly response to a question on the global satisfactory relief of abdominal bloating and sensation of flatulence by a single statement (yes or no).

### Diet registration

The patients recorded all the food they had eaten during two weekdays in the diary during the run-in and the treatment period. Nutrients were manually calculated.

### Compliance

The patients reported the daily consumption of the study product in the diary. The compliance was calculated as percent of planned ingestion of the study product, and compliance above 80 % was set as a minimum requirement in order to be regarded as acceptable.

### Tolerability and safety

Tolerability and safety were assessed by recording all reported adverse events.

### Assessment of colonic transit time

Total and segmental colonic transit time (CTT) was assessed according to Metcalf et al. [22]. The abdominal X-rays were recorded at 9:00 AM on the day after 3 days ingestion of 20 differently shaped radioopaque markers at 9:00 AM each day (Marquat Genie Biomedical, Boissy-St Léger, France). The X-rays were taken using a rapid high-kilovoltage technique to reduce radiation exposure to less than 0.5 mSv. The markers located to the right of the vertebral spinous process and above an imaginary line running from the fifth lumbar vertebra to the pelvic outlet were assigned to the right colon; those left of the vertebral spinous process and above an imaginary line running from the fifth lumbar vertebra to the anterior superior iliac crest were assigned to the left colon; and those below an imaginary line running from the pelvic brim on the right to the anterosuperior iliac crest on the left were judged to be in the rectosigmoid. Total and segmental CTT (left side, right side and rectosigmoid) were calculated according to the formula: Colonic transit (hours) = 1.2 × number of markers. A Spearman correlation explored the association between Bristol stool form scale and colonic transit time and any significant change in symptoms.

### Assessment of quality of life

Before the start of the randomisation period and at the end of it, the patients answered a self-administrated quality of life questionnaire, SF-36 (validated Italian version) [23]. This is a generic measure of perceived health status, widely used in medical and health service research, that incorporates behavioural functioning, subjective well-being and perception of health by assessing eight health concepts: physical function (how patients perceive their ability to perform physical tasks), role-physical (how patients perceive their ability to fulfil their life role physically), bodily pain (how patients perceive their level of pain), general health (how patients perceive their overall health and well-being), vitality (how patients perceive their level of ‘energy’), social function (how patients perceive their ability to participate in social activities), role-emotional (how patients perceive their ability to fulfil their life role emotionally) and mental health (how patients perceive their emotional and psychological well-being). The scores on all scales range from 0 to 100, with higher scores reflecting better health.

### Statistical analysis

The primary endpoint for analysis in this study was the global satisfactory relief of bloating and flatulence. The primary analysis was a comparison by Fisher's exact test of the proportion of responders treated with symbiotic mixture vs placebo. Responders were defined as individuals whose response to the weekly global satisfactory relief questions was yes on at least 50 % of weekly assessment of the 4-week randomisation [7].

The secondary endpoints included severity of VAS scores for symptoms and bowel function scores. The daily diary was used to appraise the bowel function scores (frequency, consistency and the proportion of days with the sense of incomplete evacuation) and the severity of individual symptoms (abdominal bloating, sensation of flatulence, pain and urgency). The VAS symptom scores are rounded to the nearest integer in millimetres. The adjectival scales for stool consistency (Bristol stool form scale) were recorded as numerical values. The data on bowel function scores and VAS scores of individual symptoms were summarised weekly as the means and standard errors in each treatment group.

In the secondary analysis, the comparison of the two groups focused on contrasting week-by-week symptoms and bowel function scores during the treatment period using a repeated-measures analysis of covariance (ANCOVA) model with the corresponding value at baseline as the covariate. Age was also included as a covariate for analyses of the symptoms. ANCOVA was used to account for the potential effects of differences in the mean baseline measurements and scores between the two treatment groups [7].

The remaining total and segmental CTT and eight-item SF-36 scores were compared within groups with paired *t* test or Wilcoxon signed-rank test for paired comparison samples, as warranted. Post-treatment between group values were computed based on two-sample *t* test or Mann–Whitney U (M-W) for unpaired comparisons, as warranted.

Data are presented as mean (±standard error (SE)), unless otherwise indicated; Significance was expressed at *p* < 0.05 level. The SPSS software package for Windows (release 18.0; SPSS Inc, Chicago, IL, USA) was used for statistical analysis.

From previous clinical trials [24, 25] that considered the intention to be able to detect a 30 % difference in the proportion of responders between the two groups with 80 % power at *a* = 0.05, a minimum of 60 patients would be required. Our study was designed to include 64 patients, 32 per treatment group. Knowing that this was a very optimistic calculation based on previous clinical trials, we also planned to perform sample size calculations for future studies based on the proportion of responders in our treatment group.

## Results

### Demographic characteristics

A total of 83 IBS patients were enrolled to obtain the calculated sample size (*n* = 64). During the run-in, 15 IBS patients were not subsequently randomised: ten withdrew their consent, one had concomitant acute diseases (thrombosed external haemorrhoids), one underwent PPI therapy, two failed to fulfil the severity criteria for bloating or flatulence and one was positive to the pregnancy test 48 h before the first transit study.

**Fig. 2:**
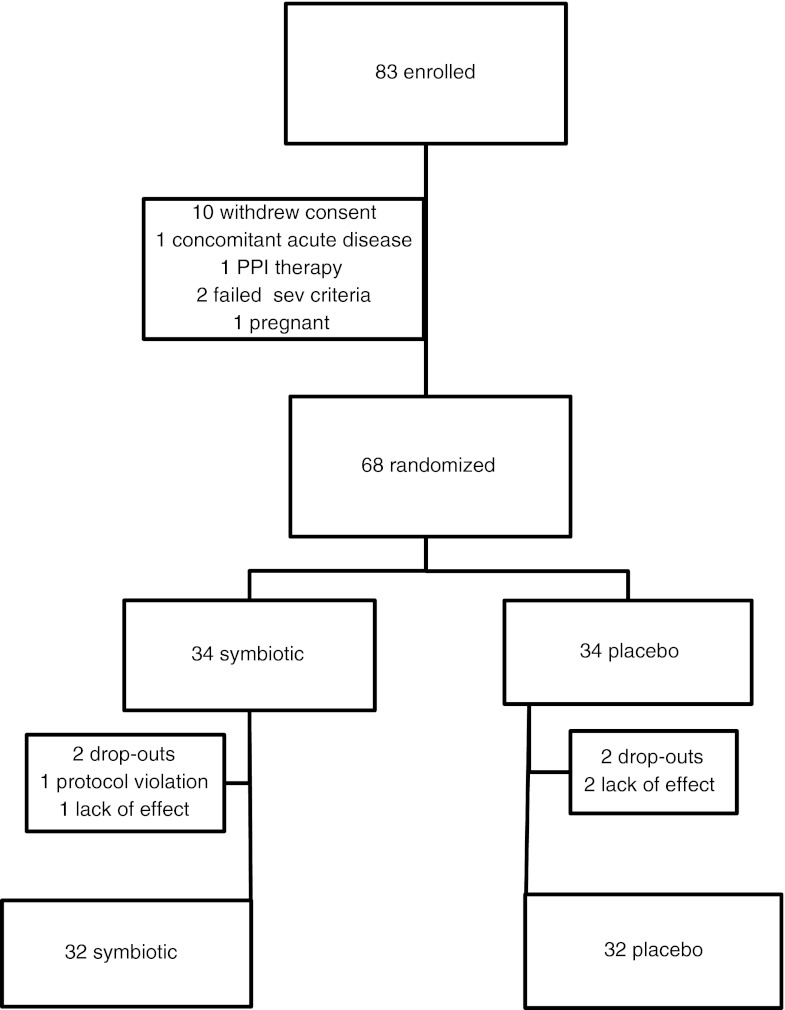
Flow chart demonstrating the number of patients in the different phases of the study

During the randomisation period, four drop-outs occurred (Fig. 2). All patients were replaced to obtain the calculated sample size.

No differences were found in age, gender, FBDSI and predominant bowel habits between symbiotic and placebo groups. At baseline, the bloating score and the sensation of flatulence were similar in both groups (Table 1).

**Table 1:** Demographic and clinical parameters of IBS population studied (*n* = 64)

	Symbiotic	Placebo
*N*	32	32
Gender, *n* (%)	22 (69)	19 (59)
Age	36.6 ± 2.2	40.8 ± 2.2
FBDSI	61.5 ± 6.9	63.7 ± 7.3
Predominant bowel habit		
Diarrhoea, *n* (%)	9 (28.1)	14 (43.8)
Constipation, *n* (%)	13(40.6)	12 (37.5)
Mixed, *n* (%)	10 (31.3)	4 (12.5)
Undetermined, *n* (%)	0 (0.0)	2 (6.3)
Bloating VAS score	40.7 ± 3.7	42.6 ± 2.8
Flatulence VAS score	36.4 ± 2.9	35.4 ± 2.7

Values are expressed as mean ± SE

### Symptoms and bowel function

#### Primary outcomes

Responders for abdominal bloating were 46.9 % (15/32) for the symbiotic group vs 65.6 % (21/32) for the placebo group (Fisher's exact test, *p* = 0.21) and for sensation of flatulence were 50 % (16/32) for the symbiotic group vs 62.5 % (20/32) for the placebo group (Fisher's exact test, *p* = 0.45).

#### Secondary outcomes

**Fig. 3:**
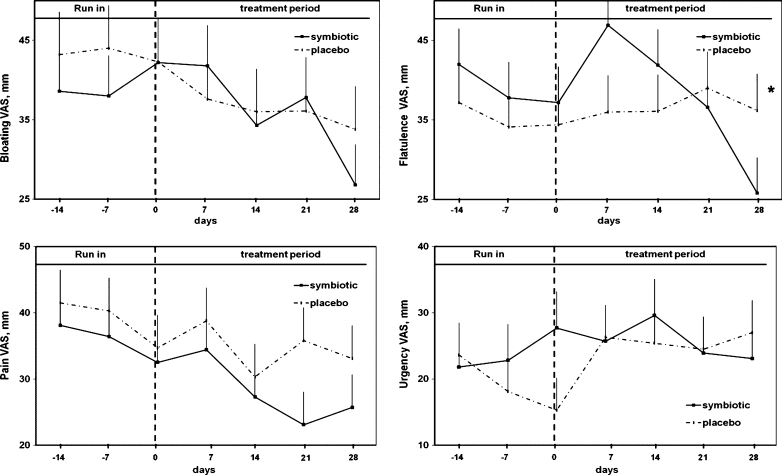
VAS scores of bloating, flatulence, abdominal pain and urgency during the run-in and treatment period in symbiotic and placebo groups. (Mean values ± SE; repeated-measures ANCOVA, *p* < 0.05)

The score of flatulence was significantly lower with symbiotic mixture compared to placebo during the week-by-week comparisons of treatment period (repeated-measures ANCOVA, *p* = 0.038). The symptoms of bloating, pain and urgency did not show any significant difference in the two groups during the treatment period (Fig. 3).

There were no statistically significant differences detected for bowel function during the week-by-week comparisons of treatment period (repeated-measures ANCOVA, frequency *p* = 0.126, consistency *p* = 0.436 and proportion of days with the sense of incomplete evacuation *p* = 0.552).

### Diet

Quality and quantity of food intake were unchanged along the entire study: run-in and randomisation period (data not shown).

### Compliance

The compliance was >95 % in both groups as analysed from the study diaries. Five of the 64 participants (one in placebo and four in the treated group) required antibiotic therapy during the study period for infection (cystitis, upper respiratory infection and prophylaxis for dental work) and adhered to protocol instructions. One patient from each treatment group used a loperamide tablet (average use, two tablets; range 1–3) as predefined rescue medication for severe diarrhoea during the 6-week trial. Other medications were: milk of magnesia (two patients for 1 day), paracetamol (two patients for 2 days), prednisone (one patient for 5 days), budesonide inhaler (one patient for 6 days) and ketoprofen (one patient for 1 day).

### Tolerability and safety

There were no adverse events noted with the use of this symbiotic mixture and/or placebo treatment.

### Colonic transit time

**Fig. 4:**
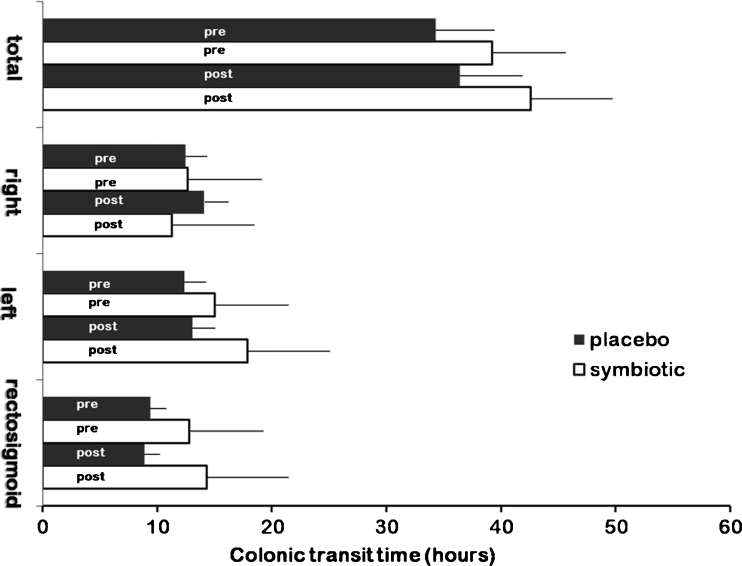
Total and segmental colonic transit time in patients with irritable bowel syndrome pre- and post-treatment with symbiotic mixture or placebo (mean values ± SE)

In the run-in period, no significant differences were found in total, right, left and rectosigmoid transit time (M-W, *p* = 0.4, *p* = 0.9, *p* = 0.5 and *p* = 0.3, respectively). Total and segmental CTT did not change from pre-treatment to post-treatment period within symbiotic and placebo group (Wilcoxon signed-rank test for paired comparisons; Fig. 4).

In post-treatment period, symbiotic group compared to placebo group showed a longer rectosigmoid transit time that just failed to reach statistical significance (symbiotic 14.3 ± 2.5 vs placebo group 8.9 ± 1.9 h (mean ± SE), M-W, *p* = 0.06), while no differences were found in total, right and left CTT.

A significant Spearman correlation (Rs) was found among total transit time and stool consistency (Bristol Scale score) at run-in and at the end of the study (Rs = −0.37, *p* = 0.003 and Rs = −0.32, *p* = 0.02). No significance was found in the Spearman correlation between the change of flatulence score (baseline to last week of treatment) and the change in total colonic transit time (baseline to end of treatment; Rs = 0.03).

### Quality of life

**Fig. 5:**
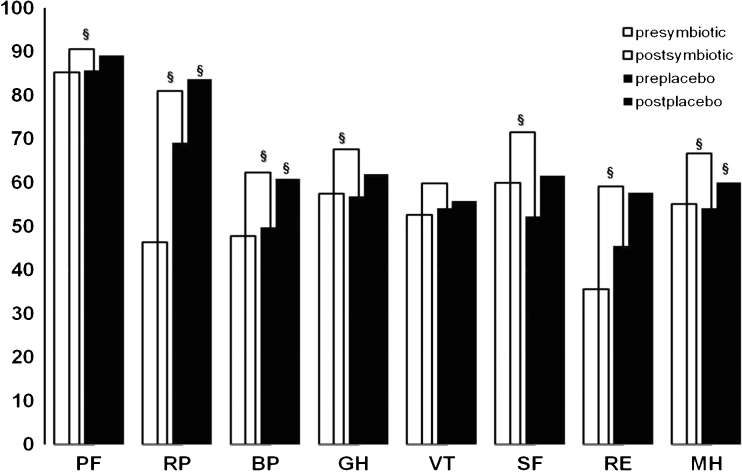
The mean SF-36 scale scores for IBS patients who underwent symbiotic mixture or placebo treatment (mean values ± SE). § *p* < 0.05 pre- vs post-treatment using paired *t* test

Figure 5 shows the eight-item SF-36 scores for symbiotic and placebo group pre- and post-treatment. All items of the SF-36 questionnaire, a part vitality domain, significantly improved from pre-treatment to post-treatment period within symbiotic group, while in placebo group, only three domains, role-physical, bodily pain and mental health, reached a significant improvement from pre-treatment to post-treatment period. However, none of the changes observed differed significantly between the groups.

## Discussion

This is the first randomised, double-blinded, placebo-controlled study investigating this particular commercially available symbiotic mixture in patients with IBS. Our results demonstrated a significant beneficial effect of the symbiotic mixture in decreasing the severity of flatulence in IBS patients. At the end of the treatment study, an increased colonic transit time in rectosigmoid and a significant improvement in most SF-36 scores were observed in IBS patients, who ingested the symbiotic mixture. However, there were no significant differences in the predefined primary endpoints, focused on the global satisfactory relief of bloating and flatulence. The symbiotic mixture and the placebo product were both well tolerated by the participants in the study, with no adverse events. IBS is a heterogeneous disorder, consisting of a number of bothersome symptoms that we are presently not able to treat efficaciously.

As recently shown, the most bothering symptom after abdominal pain is bloating, including abdominal distension and gas. Their presence is a frequent reason to seek medical care [26] and is often associated with a reduced quality of life. Several studies targeting the intestinal microbiota for treatment of functional GI symptoms reported a prominent beneficial effect of these interventions on bloating symptoms and the sensation of flatulence [6–8].

In recent years, the interest on the efficacy of probiotics in IBS led to extensive systematic reviews and meta-analyses studies focused on this topic [9–11, 27, 28]. One of these systematic reviews analysed a total of 16 randomised controlled trials and found that *B. infantis* was the only strain that could show any positive effect upon the symptoms, in two appropriately designed studies [10]. Another one, analysing the continuous data, demonstrated that combinations of probiotics improved symptoms in patients with IBS, and in addition, higher quality studies reported a more modest treatment effect, compared to lower quality studies [11].

Our finding, of a significant decrease in the severity of flatulence in IBS patients treated with the symbiotic mixture compared to the IBS patients treated with the placebo over a 4-week period of treatment, suggests a beneficial effect of this particular combination of prebiotics and probiotic in IBS patients. However, contrary to our hypothesis, the present study was not able to show an improvement of other investigated outcomes, especially those of the primary outcome (global satisfactory relief in symptoms of bloating and flatulence). This result may be related to the intrinsic limitations of the study design which was the first study on this symbiotic mixture, with a small number of patients that leads to the lack of statistical power (type 2 error) as well as to the inclusion of a heterogeneous IBS population. The primary outcome, furthermore, is a multidimensional outcome that is influenced by a number of factors, including coping mechanisms and psychological status, which may not be directly related to symptoms severity [29]. It is noteworthy that most domains in the SF-36 QoL questionnaire have improved in the active group with symbiotic treatment but only a few in the placebo group.

There are no current studies available that could prove unambiguously the mode of action of probiotics, which can be clearly linked to the improvement of IBS and its symptoms. As an attempt to investigate the possible effects of the symbiotic mixture on IBS symptoms, transit time was studied. At the end of treatment, patients treated with the symbiotic mixture showed a lengthening in rectosigmoid transit time that just failed to reach a statistical significance. The colonic transit time significantly correlated with the increase in stool consistency measured by Bristol Scale score. This may result in reduced aborad movement of stool and, possibly, the reduced flatulence [7].

Although it seems biologically plausible that modification of the gut flora could have an effect on IBS-related symptoms such as the flatulence sensation by modifying gas production (colonic milieu) and gut transit, further studies are needed to explore the mechanism of the observed retarded transit of stool and the potential effects on colonic sensation and colonic fermentation of nutrients reaching the colon. In the literature, several mechanisms have been suggested to explain the efficacy of probiotics in IBS, such as the influence of intestinal luminal environment, the maintenance of epithelial and mucosal barrier function and the modulation of mucosal or systemic immune system including both innate and adaptive immune systems [30–32]. Although it might be too early to make a conclusion of whether these mechanisms could be adapted to the pathogenesis of IBS, there is, however, a growing body of evidence that supports the association of gut inflammation/immunity and IBS and that define the roles of probiotics in the pathogenesis of IBS. Another important issue is that in the symbiotic mixture administered in the present study, there are also prebiotics and their exact role in IBS remains unclear due to the paucity of trials evaluating their treatment efficacy [33]. Prebiotics, at least in theory, may globally enhance the functions of probiotics, augment the effects of beneficial commensal bacteria of the gut and assist in the creation of an inhospitable environment for the proliferation of pathogenic bacteria [34]. As shown in Fig. 3, it is interesting that flatulence seems to worsen initially in the symbiotic group and then it improves. This is potentially an important point of interest. In fact, prebiotic, such as inulin, could initially cause an increase in flatulence delivering, as it does, unabsorbed carbohydrate to colonic bacteria. However, it is already known that colonic bacteria adapt to new substrates and will metabolise them more effectively over time, showing the beneficial effects of reducing flatulence as the colonic flora adapts.

To our knowledge, there is another randomised, double-blinded, placebo-controlled study that investigated a symbiotic mixture in IBS [15], but it studied only a single-strain probiotic and the placebo consisted of the same mixture with inactivated probiotic, leading to a possible misinterpretation of the results for a role due to the prebiotic. Although we did not perform any microbiological analyses of the stools in the present study, it has been recently shown that this particular symbiotic was able to modify the gut flora in healthy volunteers compared to placebo [35].

There are several limitations of this study. First of all, the study population was not sufficiently large enough for a subgroup analysis of IBS subtypes. We were, therefore, unable to show whether some IBS subtypes would actually benefit more from the consumption of the symbiotic mixture than the others. Additionally, the duration of the study was perhaps too short. We chose a 4-week period of treatment on the assumption that strict exclusion criteria for concomitant medications should be followed to avoid potential confounders, but it would be of specific value to explore whether patients benefit more from a longer consumption of this symbiotic mixture and also whether this symbiotic mixture needs to be given on a cyclic schedule because of the temporary modification of the faecal flora [36].

In conclusion, the symbiotic mixture (Probinul) failed to achieve an improvement in primary endpoints, i.e. global satisfactory relief of abdominal flatulence and bloating. Among the secondary endpoints, it did demonstrate a beneficial effect in decreasing the severity of flatulence in IBS patients. The mixture, however, showed a lack of any adverse events and a good side-effect profile. Further studies on a larger number of patients are indeed needed to confirm whether this symbiotic mixture might be an effective treatment option in IBS.
